# Commentary: Atypical Self-Focus Effect on Interoceptive Accuracy in Anorexia Nervosa

**DOI:** 10.3389/fnhum.2017.00228

**Published:** 2017-05-05

**Authors:** Giuliana Lucci, Panayotis Patrikelis

**Affiliations:** ^1^Department of Technologies, Communication and Society, Marconi UniversityRome, Italy; ^2^Department of Neurosurgery, School of Medicine, Evangelismos Hospital, University of AthensAthens, Greece; ^3^Hellenic Centre for Neurosurgical Research “Prof. Petros Kokkalis,”Athens, Greece

**Keywords:** anorexia nervosa, interoceptive accuracy, self-focused attention, social comparison, insular cortex

The concerning mortality rate in individuals affected by Anorexia Nervosa (AN; Arcelus et al., 2011) makes this topic extremely relevant, and the need to develop new therapeutic approaches urgent. For this reason we welcome Pollatos et al.'s (2016) work.

As it is well known, AN is a psychiatric disorder mainly involving the body, i.e., body representation and perception. Among other things, individuals with AN show difficulties in interoception (Pollatos et al., 2008; Strigo et al., 2013), i.e., perception of internal bodily signals (Craig, 2003). There is a large body of literature documenting the key role of interoception in a wide range of self-related processes, from regulation of primitive bodily needs (Berthoud, 2006; Brannigan et al., 2015) to self-awareness (Seth et al., 2012; Seth, 2013), through both behavioral (Herbert et al., 2007) and emotional regulation (Füstös et al., 2013). That is the reason why improving interoceptive accuracy (IAcc) in individuals suffering from AN is so relevant.

In their work, Pollatos et al. (2016) investigated if self-focused attention may be a viable route for improving IAcc in individuals with AN, as it is in healthy individuals (Ainley et al., 2012).

In their work, the authors asked both AN and healthy individuals to perform the heartbeat perception task (Schandry, 1981), while they were looking at their own or at another (unknown) woman's face. In this way, the authors were able to compare IAcc of both groups under enhanced and decreased self-focused attention, respectively. The results showed that interoceptive accuracy during looking at their own face was lower than during looking at another woman's face in individuals with AN, whereas the opposite pattern emerged in healthy individuals. The authors speculated that in AN individual's looking at their own face could trigger avoidance of attention on both outer and inner body, thereby reducing their IAcc.

Due to a lack of information about interoceptive accuracy at the baseline, it cannot be ruled out that looking at another person's face enhanced IAcc, whereas looking at their own face reduced IAcc in individuals with AN.

We hypothesize, together with the authors, that looking at their own face in individuals with AN decreases self-focused attention because of body-related avoidance. In addition, we hypothesize that looking at another woman's face enhanced self-focused attention in AN individuals by means of social comparison processes. In other words, it can be inferred that self-focus enhances IAcc in AN as in the healthy control group, but the difference would lie in how self-focus is achieved: looking at their own face in healthy individuals and looking at another woman's face in AN individuals.

Social comparison, i.e., the comparison between the self and the others (Festinger, 1954), engages everyone continuously, but individuals with eating disorders compare themselves with others more unfavorably than healthy individuals (Troop et al., 2003).

Furthermore, viewing another woman's body evokes more negative affect and enhances limbic activity in AN than in healthy individuals. It has been hypothesized that social comparison elicits negative emotions in anorectic individuals and it is related to limbic activation (Vocks et al., 2010). Moreover, AN individuals showed similar or even higher insular activation during a task of self-other comparison, and lower insular activation during processing of images of self rather than healthy subjects (Sachdev et al., 2008; Friederich et al., 2010). As it is well known, insula has a key role in interoception, and in healthy people higher insular activation seems to correspond to higher interoceptive accuracy (Critchley et al., 2004).

Finally, quite recently Nunn and colleagues proposed “the insula hypothesis” in AN (Nunn et al., 2008, 2011), which states that the lack of integrity of the insular cortex can explain the perceptual, emotional, behavioral and cognitive profile of AN (for a review of the role of the insula in AN, see Kaye et al., 2009, 2013).

Then, taking into account (a) the results of Pollatos et al. (2016), (b) the documented insular activation in AN individuals triggered by viewing image of other women—that in turn activates social comparison processes (Sachdev et al., 2008; Friederich et al., 2010), and (C) the insula hypothesis in AN (Nunn et al., 2008, 2011), we challengingly speculate that social comparison could be used, at least initially, in the treatment of AN to enhance self-focused attention which, as shown by Ainley et al. in healthy individuals (Ainley et al., 2012), is a way to improve IAcc.

In order to challenge our hypothesis that social comparison could be an initial way to improve IAcc in AN individuals by virtue of its capacity to activate the insula, future research should investigate brain activation in both healthy and anorectic individuals during a heartbeat perception task performed while looking at their own or at another person's face.

## Author contributions

All authors listed, have made substantial, direct and intellectual contribution to the work, and approved it for publication.

### Conflict of interest statement

The authors declare that the research was conducted in the absence of any commercial or financial relationships that could be construed as a potential conflict of interest.
